# Light-Stress Response Mediated by the Transcription Factor *Kl*Mga2 in the Yeast *Kluyveromyces lactis*

**DOI:** 10.3389/fmicb.2021.705012

**Published:** 2021-07-14

**Authors:** Ilaria Camponeschi, Arianna Montanari, Marzia Beccaccioli, Massimo Reverberi, Cristina Mazzoni, Michele M. Bianchi

**Affiliations:** ^1^Department of Biology and Biotechnology ‘C. Darwin’, Sapienza University of Rome, Rome, Italy; ^2^Department of Environmental Biology, Sapienza University of Rome, Rome, Italy

**Keywords:** lipid, membrane, desaturase, ROS, fungi

## Abstract

In unicellular organisms like yeasts, which do not have specialized tissues for protection against environmental challenges, the presence of cellular mechanisms to respond and adapt to stress conditions is fundamental. In this work, we aimed to investigate the response to environmental light in *Kluyveromyces lactis*. Yeast lacks specialized light-sensing proteins; however, *Saccharomyces cerevisiae* has been reported to respond to light by increasing hydrogen peroxide level and triggering nuclear translocation of Msn2. This is a stress-sensitive transcription factor also present in *K. lactis*. To investigate light response in this yeast, we analyzed the different phenotypes generated by the deletion of the hypoxia responsive and lipid biosynthesis transcription factor *Kl*Mga2. Alterations in growth rate, mitochondrial functioning, ROS metabolism, and fatty acid biosynthesis provide evidence that light was a source of stress in *K. lactis* and that *Kl*Mga2 had a role in the light-stress response. The involvement of *Kl*Msn2 and *Kl*Crz1 in light stress was also explored, but the latter showed no function in this response.

## Introduction

Studies on yeast lipid metabolism have been mainly conducted on *S. cerevisiae* (Natter and Kohlwein, 2013): in this yeast, fatty acid (FA) biosynthesis is restricted to saturated and monounsaturated FAs (MUFAs) and essentially accomplished by acetyl-CoA carboxylase (Acc1) and FA synthase (Fas1 and Fas2) enzymes and by the unique Δ9 desaturase enzyme (Ole1) that produces the monounsaturated (MUFA) palmitoleic and oleic acids. In the lactose-utilizing yeast *Kluyveromyces lactis*, the composition of FAs is enriched with the polyunsaturated (PUFA) linoleic and α-linolenic acids generated by the Δ12 (Fad2) and ω3 (Fad3) desaturases (Kainou et al., 2006; Santomartino et al., 2017). Environmental conditions regulate the expression of desaturases and have therefore effects on the synthesis and abundance of different (mono- and poly-) unsaturated FA molecules (Rossi et al., 2009) and on properties of membranes.

Mga2 is the *S. cerevisiae* transcription factor that regulates the expression of desaturase gene *OLE1* (Zhang et al., 1999; Chellappa et al., 2001; Jiang et al., 2001) in low-oxygen conditions. Mga2 is constitutively expressed as inactive form bound into endoplasmic reticulum (ER). It is activated in hypoxic conditions by proteasome cleavage (Hoppe et al., 2000): the 90 kDa N-terminal soluble fragment moves then into the nucleus where it induces the expression of low-oxygen responsive genes. In *K. lactis*, studies on *KlMGA2* gene, which is the ortholog gene of *S. cerevisiae MGA2* and/or *SPT23*, revealed multiple cellular roles of this regulator, including response to hypoxia, respiration, glucose metabolism, response to ROS, life span, and general cellular fitness (Micolonghi et al., 2012; Ottaviano et al., 2015; Santomartino et al., 2019a). In detail, the genetic targets of *Kl*Mga2 in FA biosynthesis are *KlACC1* and *FAD2* genes and phenotypes generated by *KlMGA2* deletion are restored by the addition of unsaturated FAs (UFA).

Light is a common environmental source of heat and energy, essential for biosynthetic organisms. Yeasts, on the other hand, lack specialized light sensing proteins such as phytochromes, opsins, and cryptochromes (Idnurm et al., 2010), but studies of light-induced stress conducted in *S. cerevisiae* have shown a response to blue light *via* activation of the stress-regulated transcription factors Msn2 and Crz1. Both factors control gene expression through nucleo-cytoplasmic translocations (Cai et al., 2008; Bodvard et al., 2011), and the homologs of these two stress-sensitive transcription factors were identified also in *K. lactis* (Bussereau et al., 2006; Barsoum et al., 2011). In *S. cerevisiae*, Ca2+ signaling, mediated by the Ca2+/calmodulin dependent phosphatase calcineurin, is required for cell survival during environmental stresses. These conditions cause an increase in cytosolic Ca2+ that induces calcineurin activation (Cyert, 2003). The phosphatase, in turn, dephosphorylates Crz1, which rapidly translocates from the cytosol to the nucleus, where it activates the transcription of genes involved in adaptation to stress (Bodvard et al., 2013; Thewes, 2014).

The second stress-response transcription factor, Msn2, exhibits similar behaviors; it moves to the nucleus where it affects the expression of target genes with STREs (stress response elements) in their promoters (Estruch, 2000). Several stresses are known to induce nucleocytoplasmic oscillations of Msn2, including high concentrations of extracellular Ca2+ (Cai et al., 2008), caloric restriction (low glucose levels 0.1%) (Medvedik et al., 2007), oxidative stress (Hao and O’Shea, 2011), as well as light (Bodvard et al., 2011, 2013, 2017), but the overall activation pathway is less clear than Crz1. Nuclear localization of Msn2 is contrasted by cyclic AMP-controlled protein kinase A (PKA) (Görner et al., 1998) and promoted by the phosphatases PP1 and PP2A (Görner et al., 1998; Santhanam et al., 2004). Another important feature is that light stimulates hydrogen peroxide production in cultured mouse, monkey, and humans cells *via* photoreduction of flavin-containing oxidases (Hockberger et al., 1999). The intermediates and the mechanism by which PKA senses light remain unclear, but it has been shown that in *S. cerevisiae* a conserved peroxisomal oxidase (Pox1) converts light into a H_2_O_2_ signal, which is sensed by the peroxiredoxin Tsa1 and then transduced to thioredoxin (Trx1) to inhibit PKA activity (Bodvard et al., 2017).

To our knowledge, studies on response to light have never been reported in *K. lactis* except the resonance response to light–dark 12 + 12 hour cycles generating an increase of phenotypic suppression in *KlMGA2* deletion mutant (Camponeschi et al., 2020). Interestingly, the desaturase gene *FAD2* is a light-dependent gene in plants (Kargiotidou et al., 2008; Dar et al., 2017) and its transcription is increased also at low temperatures (Byfield and Upchurch, 2007). Desaturase level and lipid unsaturation index were both light-dependent in cold stress (Yuan et al., 2012). As reported above, in *K. lactis FAD2* gene is a hypoxic target of *Kl*Mga2, suggesting that it could be possible for an overlap between light response and other conditions affecting the regulation of FA biosynthetic metabolism, such as hypoxia, also in non-photosynthetic organism.

In this work we aimed to investigate the role of the yeast multi-functional mediator *Kl*Mga2 in light response and connection with the other putative stress factors *Kl*Msn2 and *Kl*Crz1 in this yeast. Effects of light on oxidative stress and lipid biosynthesis were also studied.

## Materials and Methods

### Media and Growth Condition

The yeast strains used are listed in Table 1. The YPD medium was composed of 1% Yeast Extract (Becton Dickinson and Company), 1% Peptone (Becton Dickinson), and 2% glucose. Solid media contained 2% Bacto-Agar (Becton Dickinson). For selection of transformed cells, we used YPD solid medium supplemented with Geneticin 100 μ/ml (G418; Sigma-Aldrich) or SD solid medium, composed of 0.67% Yeast Nitrogen Base (Becton Dickinson), 2% glucose, and auxotrophic requirements as needed, without uracil. GAA medium, used to select rag + phenotype cells, was composed of YP medium with 5% glucose and 5 μM Antimycin A (Sigma-Aldrich). Except when otherwise specified, culture growth was performed at 28 ± 1°C in complete darkness or in light (134 μM/s/m^2^) under white LED lamps (4500K, 400–700 nm range). Cell density was determined by optical density at 600 nm or by cell counting in Burker chamber.

**TABLE 1:** Plasmids and strains.

Name	Description	References
**Plasmids**		
pFA6-KanMX4	Plasmid containing the *KanMX4* marker	Wach et al., 1994
pYM27	Template for C-terminal EGFP and G418^R^	Janke et al., 2004
pYM14	Template for C-terminal 3HA and G418^R^	Janke et al., 2004
Kp426[KlMga2^FLAG^]	Plasmid containing Flag-tagged *KlMGA2*	Micolonghi et al., 2012
pRS416	Plasmid containing the *URA3* marker	Sikorski and Hieter, 1989
**Strains**		
MWL9S1	*MAT*a, *ura3*, *leu2*, *lysA1*, *trp1*, *metA1-1*, *Klnej1::loxP*	Hnatova et al., 2008
*Klcrz1*Δ	*MAT*a, *ura3*, *leu2*, *lysA1*, *trp1*, *metA1-1*, *Klcrz1::kanMX4*	This work
*Klmsn2*Δ	*MAT*a, *ura3*, *leu2*, *lysA1*, *trp1*, *metA1-1*, *Klmsn2::kanMX4*	This work
LDA2	*MAT*a, *ura3*, *leu2*, *lysA1*, *trp1*, *metA1-1*, *fad2::kanMX4*	De Angelis et al., 2016
LD2G	*MAT*a, *ura3*, *leu2*, *lysA1*, *trp1*, *metA1-1*, *FAD2-GFP*	De Angelis et al., 2016
*Klmga2*Δ	*MAT*a, *lysA1*, *trp1*, *leu2*, *metA1-1*, *ura3, Klnej1::loxP*, *Klmga2::URA3*	This work
*mga2*ΔTM	*MATa*, *lysA1*, *trp1*, *leu2*, *metA1-1*, *ura3, Klnej1::loxP*, *Klmga2*(ΔTM)-*3HA*	This work
KlMga2^FLAG^	*MATa*, *lysA1*, *trp1*, *leu2*, *metA1-1*, *ura3*, *Klnej1::loxP*, *Klmga2::URA3*, transformed with Kp426[KlMga2^FLAG^]	This work
*Klmga2*Δ*/*Fad2-GFP	*MATa*, *lysA1*, *trp1*, *leu2*, *metA1-1*, *ura3*, *Klnej1::loxP*, *Klmga2::URA3*, *FAD2-GFP*	This work

### Construction of Strains

Strains MWL9S1/*crz1*Δ, MWL9S1/*msn2*Δ, and MWL9S1/*Klmga2*Δ were obtained by disruption of *KlCRZ1* (ORF *KLLA0E08713g*), *KlMSN2* (ORF *KLLA0F26961g*), or *KlMGA2* (ORF *KLLA0E17953g*) with the *KanMX4* cassette or *URA3* deletion cassettes, which have been generated by the short flanking sequences-PCR method (Janke et al., 2004) (Vent DNA Polymerase; New England Biolabs). DNAs were amplified using the primers 1–6 (Supplementary Table 1) and plasmid pFA6-KanMX4 (Addgene, Table 1) as DNA template containing *KanMX4* or the plasmid pRS416 (Stratagene, Table 1) as DNA template containing *URA3* marker. The purified PCR products were then used to transform MWL9S1 strain by electroporation. Transformed colonies were selected on YPD solid medium supplemented with Geneticin (G418) on SD solid medium without uracil and then analyzed by PCR (Taq Pol, Jena Bioscience). Strain MWL9S1/*mga2*ΔTM was obtained in the same way by disruption of C-terminal membrane-anchoring domain coding sequence in *KlMGA2* (from codon 833 of protein KLLA0E17953p) using plasmid pYM14 (Euroscarf, Table 1) as template bearing the *3HA-KanMX4* cassette (primers 7 and 8, Supplementary Table 1). *FAD2* gene was fused in frame with *GFP* sequence in *Klmga2*Δ strain, by a PCR-based strategy (Janke et al., 2004) using plasmid pYM27 (Euroscarf, Table 1) as template bearing the *EGFP-KanMX4* cassette to obtained *Klmga2*Δ/Fad2-GFP strain. Yeast strains were obtained by transformation with the electroporation procedure as previously described (Salani and Bianchi, 2006).

### RNA Extraction and Analysis

Total RNAs were prepared by the hot phenol procedure as described by Köhrer and Domdey (1991) from cultures grown to OD_600_ = 1 ± 0.2 in light or dark conditions, and then collected or subjected to the opportune light/dark shift. Integrity of total RNAs extracts was controlled by electrophoresis on agarose-formaldehyde gel. RNA concentration was determined by measuring the absorption at 260 nm. Transcript analysis was performed by qRT-PCR. Total RNA (1 μg) was retro-transcribed to cDNA using QuantiTect Reverse Transcription Kit (Qiagen), following instructions. One sample not retro-transcribed was used as negative control. cDNA amplification was performed using KAPA Sybr Fast 2X (Sigma-Aldrich) with the appropriate couple of 10 μM primers (Supplementary Table 1) and 2 μl of the cDNA (100 ng). All the samples, except for the negative controls, were loaded in double for a technical replicate. The experiments were then conducted using the Rotor Gene Q (Qiagen), and data were analyzed using the provided software. The cycling was set with 3 min at 95°C for enzyme activation, then 40 cycles at 95°C for 3 s and 30 s at 60°C, followed by a final step for generation of melting curve, ranging from 60° to 95°C. Quantification was performed by the construction of a standard curve with genomic DNA from MWL9S1 with five points of serial dilutions (the R2 obtained for the curve was always higher than 0.99) and by the relative quantification of the samples of interest, using amplification of 18S rDNA as reference (Santomartino et al., 2019b).

### Protein Extraction and Western Blotting

Cultures were grown to OD_600_ = 1 ± 0.2 and then collected or shifted to light or dark conditions for different time points, depending on the experiment. Cells were harvested and suspended in 200 μl of sterile water. An equal volume of 0.2 N NaOH was added and cells were incubated at room temperature for 5 min, then centrifuged for 2 min at 10,000 g. After elimination of the supernatant, pellets were suspended in Laemmli buffer (60 mM TrisHCl pH 6.8; 50% glycerol; 2% SDS; 5% β-mercaptoethanol; 2% bromophenol blue). Total protein extract amount was quantified by measuring absorption at 280 nm (NanoDrop^TM^ 2000 spectrophotometer, Thermo Fisher Scientific). The samples were boiled for 5 min or incubated at 65°C for 10 min, kept on ice for 2 min and centrifuged at 10,000 g for 1 min, then loaded on 8–10% acrylamide (Sigma-Aldrich) gel for SDS-PAGE. After electrophoresis, proteins were transferred to PVDF transfer membrane (Merck Millipore). Western blotting was performed with different primary antibodies (sc-7392 anti-HA Santa Cruz Biotechnology; sc-9996 anti-GFP Santa Cruz Biotechnology; F4042 anti-flag Sigma-Aldrich) and horseradish peroxidase-conjugated secondary antibody (sc-516102 anti-mouse Santa Cruz Biotechnology). Detection was performed with ECL Western blotting detection reagents (LiteAblot EXTEND, EuroClone or Western Bright Quantum, Advansta) and visualized by ChemiDoc^TM^ MP Imaging System (Biorad). α-Tubulin (sc-53030 Santa Cruz Biotechnology) or Ada2 (ab215524 AbCam) detections were used as loading controls.

### Catalase and Superoxide Dismutase Activity

Cells (3–4 OD_600_ units) were collected from cultures, and extracts were prepared by glass beads crushing in lysis buffer (50 mM Tris-HCl pH 6.8; 100 mM NaCl). Protein content in samples was determined at 280 nm (NanoDrop^TM^ 2000, Thermo Fisher Scientific). To determine catalase activity, 1–1.5 μl aliquots of samples were added to 0.5 ml of 11 mM H_2_O_2_ (Sigma-Aldrich) in 50 mM phosphate buffer pH 7.0; 1 μM EDTA. H_2_O_2_ decomposition was monitored at 25°C at 240 nm (ε_240_ = 39.4 M^–1^ cm^–1^). Superoxide dismutase activity was determined by measuring the rate of WST1-Formazan formation, using the SOD Assay kit-WST (Sigma-Aldrich), as suggested by the supplier. Calibration curve was determined using a commercial Sod enzyme (Sigma-Aldrich). Activity measurements have been performed in two to four biological repetitions, each of them in technical triplicates. Standard deviations and statistical significances (*P*-values) have been determined.

### Fluorescence Microscopy

Exponentially growing cells on YPD medium were observed with a Zeiss Axio Imager Z1 fluorescence microscope with an AxioVision 4.8 digital image processing system, and oil 63× objective lens. Culture (100 μl) was centrifugated, washed with sterile water, and then resuspended in 100 μl of 0.1 mM 2-(p-dimethylaminostryryl)-l-methylpyridinium iodine (DASPMI). Fluorescence could be detected 5–10 min after addition of the dye to the cells. The fluorescence was observed using DASPMI filter sets (550/25 nm excitation and 605/670 nm emission). DASPMI maximal absorption wavelength is 429 nm, and maximal emission wavelength (excitation at 467 nm) is 557.5 nm in water (Bereiter-Hahn, 1976; Ramadass and Bereiter-Hahn, 2008).

### Respiration

Respiration was analyzed by measuring the oxygen consumption rate using a Clark oxygen electrode (Hansatech Instruments) as described in De Luca et al. (2009). Cells (1 × 10^6^) from exponential cultures (1 ÷ 4 × 10^6^ cells ml^–1^) were collected, washed with 1 ml sterile water, suspended in 1 ml sodium phosphate buffer (10 mM pH 7.4, containing glucose 4 g L^–1^), and loaded in the reaction vessel of the previously calibrated oxygen electrode chamber.

### Fatty Acid Extraction and HPLC-MS/MS Analysis

FAs were extracted from cells grown to OD_600_ = 1 in light or dark exposition. Lyophilized cells of *K. lactis* were extracted following the method described in Ludovici et al. (2014). Internal reference standard was the 9(S)-HODE-d4 (Cayman) at the final concentration of 1 μM. The samples were extracted with 2 ml of isopropyl alcohol:water:ethyl acetate (1:1:3 v/v) mixture with 0.0025% w/v of butylated hydroxytoluene. The extracts were dried by nitrogen flux and resuspended with 100 μl of methanol. The samples have been analyzed with LC (HPLC 1200 series rapid resolution) coupled to a triple quadrupole MS (G6420 series triple quadrupole, QQQ) equipped with an electrospray ionization source (ESI). The equipment, the chromatographic column, and the analysis software were from Agilent Technologies. The chromatographic separation has been performed with a Zorbax ECLIPSE XDB-C18 rapid resolution HT 4.6 × 50 mm 1.8 μm p.s. column. FAs were analyzed by single ion monitoring (SIM) method in negative. The elution program requires the following mobile phase: phase A water/acetonitrile 97:3 v/v containing 0.1% formic acid and 3% acetonitrile, and phase B: acetonitrile/isopropyl alcohol 90:10 v/v. The injection volume was 10 μl. Instrument setting was reported previously (Ludovici et al., 2014). The SIM parameters have been obtained by flow injection of authentic standard and agreed with the literature (Yang et al., 2009; Beccaccioli et al., 2021). FA mass and relative parameters of analysis are reported in Table 2. SIM data have been processed using Mass Hunter Quantitative software. Membrane fluidity, expressed as the FA unsaturation index (UI), was calculated as follows: [(%C16:1 + %C18:1) + (%C18:2 × 2) + (%C18:3 × 3)]/100.

**TABLE 2:** Fatty acid SIM method.

Compound name	Ion mass [M-H]^–^	Fragmentor (V)	Polarity
16:0	255.2	140	Negative
16:1	253.2	140	Negative
18:0	283.2	140	Negative
18:1	281.2	140	Negative
18:2	279.2	140	Negative
18:3	277.2	140	Negative

## Results

### Growth of *KlMGA2*, *KlCRZ1*, and *KlMSN2* Mutant Strains in Light or Dark Condition

In order to test the role of *Kl*Mga2, *Kl*Msn2, and *Kl*Crz1 in light-dependent response, we constructed deletion mutants into *K. lactis* MWL9S1 strain (Wésolowski-Louvel, 2011). The identification of *KlMGA2* has been described previously (Micolonghi et al., 2012). The homologs of *CRZ1* and *MSN2* were identified in *K. lactis* genome using BLAST: *KlMSN2* (ORF *KLLA0F26961g*) had 24% identity and 35% similarity with *MSN2*; *KlCRZ1* (ORF *KLLA0E08713g*) had 28% identity and 40% similarity with *CRZ1* (Bussereau et al., 2006; Barsoum et al., 2011). These mutant strains were named *Klmga2*Δ, *Klcrz1*Δ, and *Klmsn2*Δ, respectively. Likewise, we generated an additional *Klmga2*Δ mutant strain harboring a truncated form of *KlMGA2* (*mga2*ΔTM strain): the latter contained *Kl*Mga2 lacking C-terminal transmembrane domain (TM), that in *S. cerevisiae* represses Mga2 protein activity (Chellappa et al., 2001; Huang and Kao, 2018). We assumed that the *mga2*ΔTM strain coded for an always active truncated *Kl*Mga2 protein. Genotypes of these four mutant strains, obtained as described in the “Materials and Methods” section, are reported in Table 1.

**FIGURE 1:**
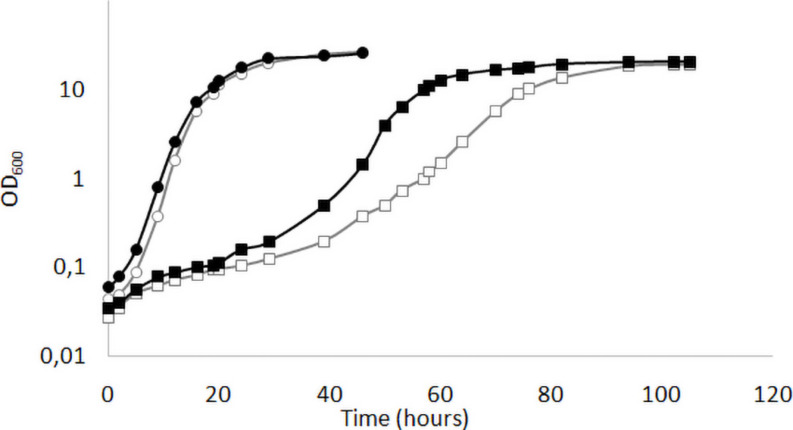
Growth profiles of *Klmga2*Δ strain in light or dark exposure. Typical growth curves of the wild type MWL9S1 (circles) and the *Klmga2*Δ mutant strains (squares) in standard YPD medium are reported as optical density (OD_600_) vs. time (hours). Black and white symbols refer to darkness and light cultivation, respectively. Repeated experiments gave similar results.

We have previously shown (Ottaviano et al., 2015) that the deletion of *KlMGA2* in the wild type GDK strain caused a reduced growth rate compared to wild type. We performed the growth experiment on standard glucose medium in light or darkness condition with MWL9S1 strain and the derived *Klmga2*Δ mutant strain (Figure 1). According to our previous results with the mutant strain GDK/*Klmga2*Δ, we observed that the deleted strain presented a longer lag phase and a slower growth rate as compared to wild type MWL9S1. Interestingly, incubation in dark or light conditions significantly affected the growth rate of mutant strain: in fact, the duplication time of deleted strain in the exponential phase shifted from about 6 h in light to 4 h in darkness, while the wild type duplication time only changed from 2.7 h in light to 2.2 h in darkness condition, respectively.

Previous studies showed that the desaturase gene *FAD2* is a target of *Kl*Mga2 (Ottaviano et al., 2015). However, light or dark incubation did not interfere with growth rates of *fad2*Δ strain (LDA2 strain, Table 1). The presence of light or darkness did not interfere significantly also with strain *Klmsn2*Δ: both results are reported in Supplementary Figure 1.

### Effects of Light and Dark Exposure on Respiration and Mitochondrial Morphology

**FIGURE 2:**
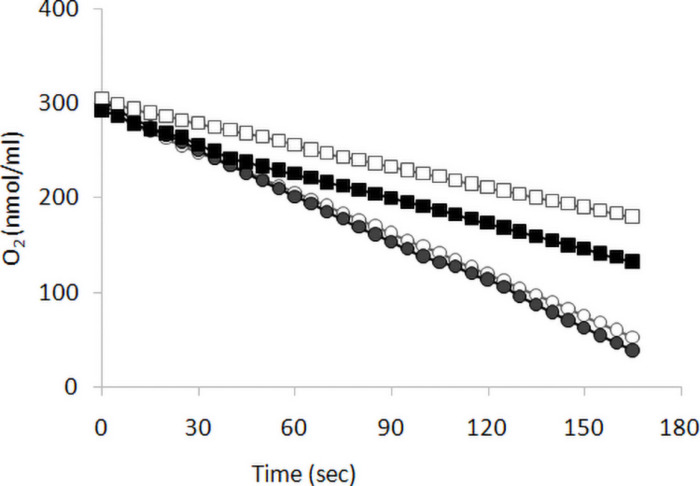
Respiration rate. Respiration was determined as oxygen consumption rate (oxygen slope: nmol O_2_ ml^–1^ s^–1^) by wild type (circles) and of *Klmga2*Δ mutant cells (squares), grown exponentially in YPD medium. Black and white symbols refer to dark and light cultivation, respectively. Repeated experiments gave similar slopes: average values and standard deviations of the repeated experiments are reported in text.

We previously reported (Ottaviano et al., 2015) that *KlMGA2* deletion affected FA biosynthesis, respiration rate, and mitochondrial morphology. To establish the occurrence of a light-dependent connection between growth and respiration rates of *Klmga2*Δ strain, we measured oxygen consumption rates of exponentially growing MWL9S1 wild type and *Klmga2*Δ mutant cells cultured in light or darkness using a Clark electrode, as described in the section “Materials and Methods.” Results are reported in Figure 2. Average of three independent measurements indicated that the wild type cells consumed oxygen faster than deleted strain and consumption rate was independent on light (1.5 ± 0.05 nmol O_2_ ml^–1^ s^–1^) or dark (1.54 ± 0.04 nmol O_2_ ml^–1^ s^–1^) growth. Differently, the deleted strains showed higher oxygen consumption rate when grown in darkness (0.92 ± 0.01 nmol O_2_ ml^–1^ s^–1^) than in light condition (0.69 ± 0.03 nmol O_2_ ml^–1^ s^–1^). Similarly to growth rate, the defective respiration rate of the deleted strain was exacerbated by light exposure while no effect of light could be recorded in the presence of *Kl*Mga2. Data reported in Supplementary Figure 2 showed that light or dark did not affect the respiration rates of *Klmsn2*Δ and *fad2*Δ strains.

**FIGURE 3:**
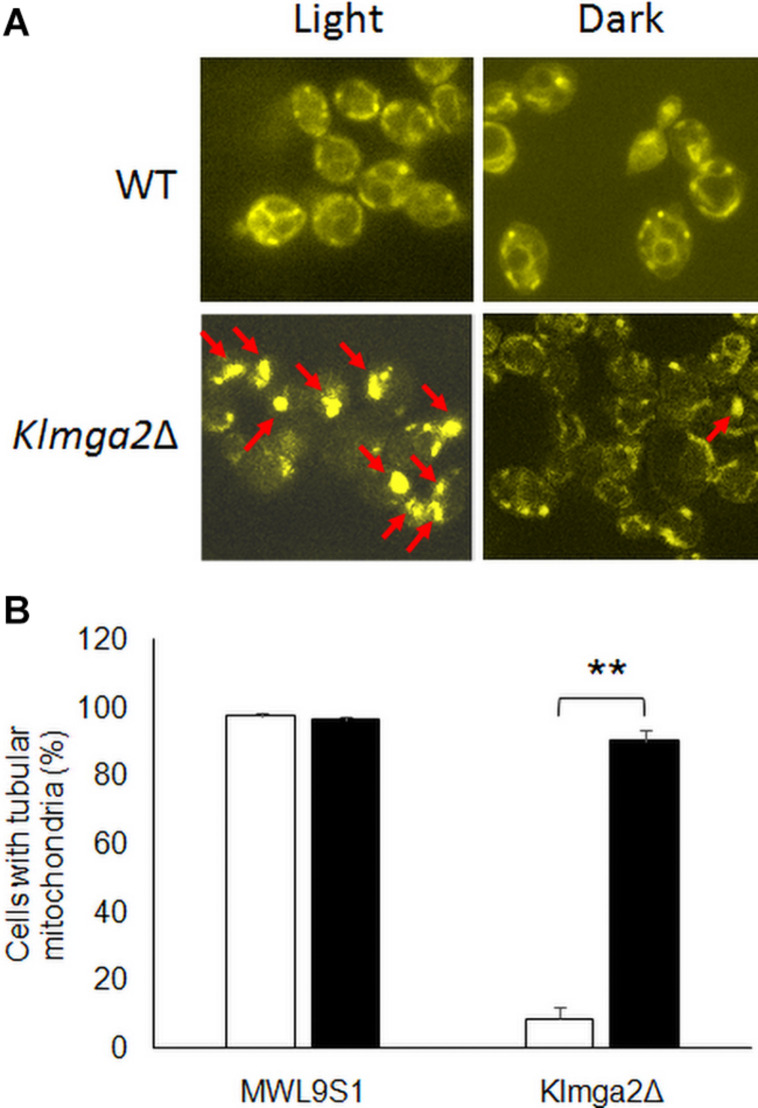
Fluorescence microscopy analysis of wild type and mutant strains *Klmga2*Δ. **(A)** Reports DASPMI staining of the mitochondrial network of wild type MWL9S1 and *Klmga2*Δ strains. The red arrows show the non-tubular collapsed mitochondrial network. **(B)** Shows histogram reporting the percentages of tubular mitochondria-containing cells. White and black blocks refer to light and darkness cultivation, respectively. Growth medium was YPD. Two or three independent cultures were performed for each strain/condition, and 100–700 cells were analyzed. Standard deviations (SD) are reported. ** indicates *p* < 0.01.

In order to investigate mitochondrial morphology and activity, cells were stained with the functional dye DASPMI. This staining allows to visualize functional mitochondrial membranes with an active potential by fluorescence microscopy (Bereiter-Hahn, 1976). Results, reported in Figure 3A and Supplementary Figure 3, revealed that the mitochondrial membranes of the *Klmga2*Δ mutant strains, as well as those of the wild type strain, were visualized by DASPMI staining suggesting a correct mitochondrial functionality. Nevertheless, as previously reported (Ottaviano et al., 2015), mitochondrial network of *Klmga2*Δ strain frequently showed a non-tubular collapsed morphology under light conditions. However, when the *Klmga2*Δ cells were grown in the darkness, the percentage of cells bearing normal tubular mitochondria was significantly increased to the same level as the wild type (Figure 3B). We observed a normal mitochondrial morphology in *Klmsn2*Δ, *fad2*Δ, and *mga2*ΔTM strains (data not shown).

**FIGURE 4:**
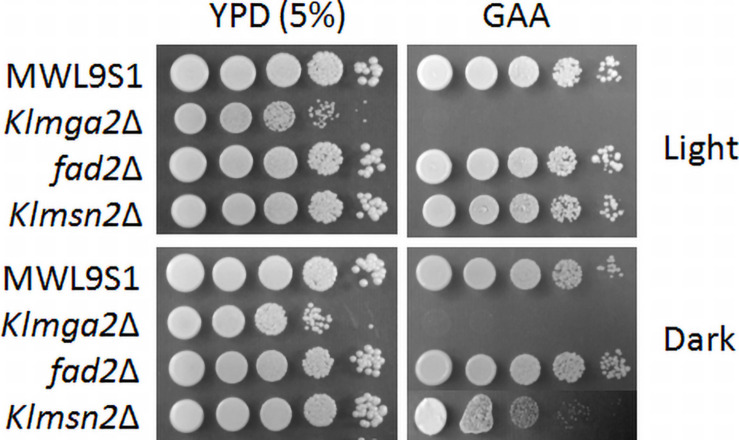
Growth on different media of wild type strain MWL9S1 and mutant strains. Cultures were grown overnight in YPD at 28°C in light or dark conditions, serially diluted, and plated onto YPD plates (5% glucose) without or with antimycin A (GAA plates). Plates were incubated at 28°C under light or dark conditions for 3–4 days. Two or three independent cultures were performed for each strain/condition. Representative samples are shown.

The *Klmga2*Δ strain in the MWL9S1 background had a rag- phenotype (sensitivity to the mitochondrial drug antimycin A on high glucose concentration: GAA medium, Figure 4) as previously reported for another *K. lactis* strain (Micolonghi et al., 2012). The rag- phenotype is usually due to defects in the glycolytic and/or fermentative pathways or in their regulation (Wésolowski-Louvel et al., 1992) pointed out by respiration blockage; however, the deletion of *KlMGA2* did not impeded hypoxic growth or ethanol production (Ottaviano et al., 2015), indicating that the inability of the deleted strain to grow when oxidative phosphorylation is impaired was not to be ascribed to mere metabolic defect. We assayed the effect of darkness on the rag- phenotype of the deleted strain (Figure 4), but no difference with light stress could be recorded, suggesting that blockage of electron transport required *Kl*Mga2 for growth in both conditions. Surprisingly, the deletion of *KlMSN2* did not affect growth in the presence of antimycin A under light stress but a slight reduction of growth was observed in darkness. Deletion of *FAD2* and *KlCRZ1* did not influence growth on antimycin A in light or darkness (Supplementary Figure 4). Expression of the *Kl*Mga2 truncated form in strain *mga2*ΔTM allowed wild type growth in the presence of light (Supplementary Figure 4); however, growth in darkness was more similar to the deleted strain than the wild type and the presence of antimycin A slightly reduced grow rate in both light and darkness.

### Activation of *Kl*Mga2 Protein

In *S. cerevisiae* Mga2 is present in two forms: p120, an inactive 120 kDa precursor form that is anchored to the ER membrane by the C-terminus, and p90, a soluble 90 kDa N-terminus fragment which is the nuclear active form. Mga2 activation requires appropriate stimuli, such as hypoxia or low temperature, to induce p120 ubiquitylation and the consequent proteolytic activation process by the proteasome. Activation of the precursor p120 releases the soluble and active transcriptional activator p90 from the ER to the nucleus, where it drives the gene expression mediated by stress responsive elements (STRE) (Covino et al., 2016).

In order to assess the role of light in KlMga2 activation, we transformed the *Klmga2*Δ strain with a plasmid containing a modified *KlMGA2*^Flag^ gene, encoding a N-terminus tagged protein, and we investigated *Kl*Mga2 protein maturation under light or dark growth conditions by Western blot. Results (not shown) revealed that *Kl*Mga2 proteolytic activation occurred independently on light or dark conditions with similar efficiency.

### Effects of Light on Catalase and Superoxide Dismutase Enzyme Production

Collapsed mitochondria are often associated to ROS accumulation and impaired response to oxidative stress: we previously observed that GDK/*Klmga2*Δ strain showed a stronger protection against ROS by over-expressing ROS detoxification enzymes catalase (Cat) and superoxide dismutase (SOD) in the exponential growth phase as compared to wild type strain (Santomartino et al., 2019a). The deletion of *KlMGA2* also caused an increased chronological life span (Santomartino et al., 2019a).

**FIGURE 5:**
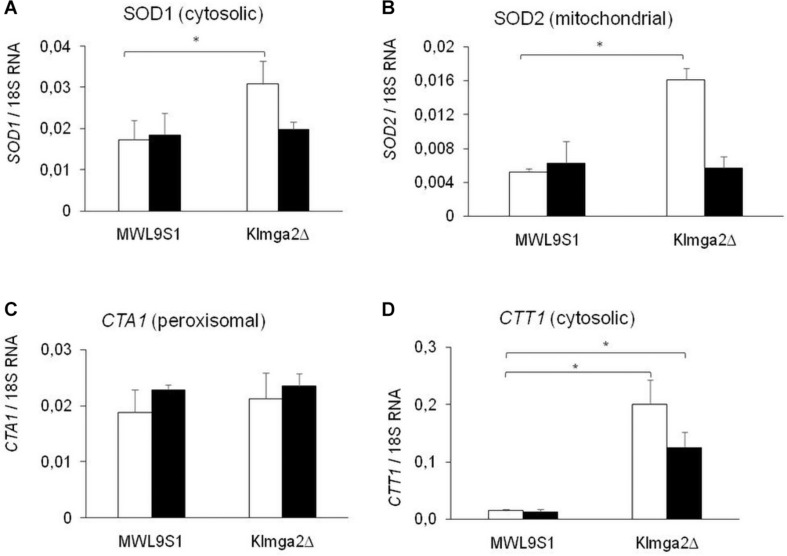
Transcription of *KlSOD1*, *KlSOD2*, *KlCTA1*, and *KlCTT1* genes. Results of qRT-PCR analysis of Catalase (Cat) and SOD genes in wild type MWL9S1 and in *Klmga2*Δ mutant strain grown in YPD flask cultures in light (white blocks) or dark (black blocks) condition are reported. **(A–D)** report *KlSOD1*, *KlSOD2*, *KlCTA1*, and *KlCTT1* transcription analysis, respectively. Ribosomal 18S gene transcription has been used as reference. Values are averages of three independent determinations, each measured by two technical repetitions, with SD reported. ^∗^ indicates *p* < 0.05.

**FIGURE 6:**
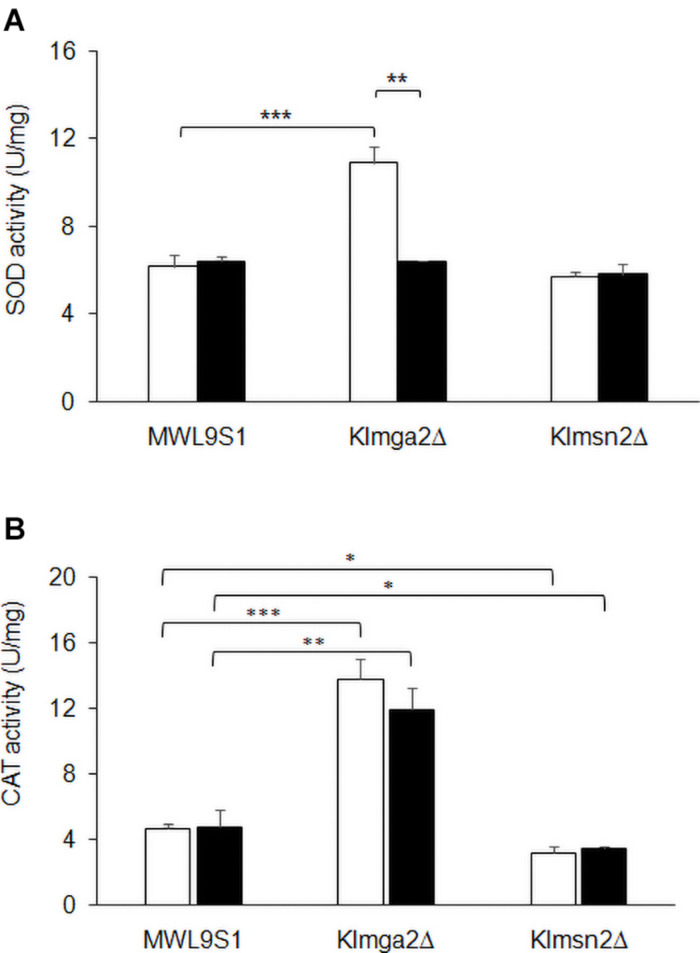
Activity of SOD and catalase enzymes. Total extracts from wild type MWL9S1, *Klmga2*Δ and *Klmsn2*Δ strains, grown in light (white blocks) and dark (black blocks) conditions up to exponential phase, were assayed for catalase and superoxide dismutase activities. **(A)** shows SOD activities (U mg^–1^): values are averages of three independent determinations, each measured by three technical repetitions, with standard deviations reported. **(B)** shows *Kl*Ctt1/*Kl*Cta1 activities (U mg^–1^): values are averages of two to four independent determinations, each measured by three technical repetitions, with SD reported. Significances: *p < 0.05; **p < 0.01; and ***p < 0.001.

Studies on *S. cerevisiae* reported that light is converted to an oxidative stress signal inside the cell (Bodvard et al., 2017). To assess if light exposition activates an oxidative stress response in *K. lactis*, we assayed the production of SOD and *Kl*Ctt1/*Kl*Cta1 enzymes by transcription analysis of *KlSOD1*, *KlSOD2*, *KlCTA1*, and *KlCTT1* genes (Figure 5) and by measuring SOD and catalase enzymes activity (Figure 6). Wild type and *Klmga2*Δ strains were cultivated in standard YPD medium in light and dark conditions to exponential growth phase. Results of transcription analysis, performed by qRT-PCR, showed that in the wild type strain transcript levels of the four genes did not change between light and dark growth condition (Figure 5). On the contrary, the transcription levels of *KlSOD1* (cytosolic SOD) and *KlSOD2* (mitochondrial SOD) genes were higher in the mutant strain than the wild type when cells were grown in light (Figures 5A,B). We also observed a 10-fold higher transcription level of *KlCTT1* (cytosolic Cat) gene in the mutant strain with respect to the wild type cells in both light and dark condition (Figure 5D). Differently, the transcription levels of *KlCTA1* (peroxisomal Cat) gene were not significantly different between *Klmga2*Δ mutant and wild type cells (Figure 5C).

To assess if increased transcription of the selected genes corresponded to increased enzyme activities, SOD and *Kl*Ctt1/*Kl*Cta1 activities were measured in cell extracts at the exponential growth phase. Results are reported in Figure 6. Both SOD and *Kl*Ctt1/*Kl*Cta1 activity levels in wild type strain resulted independent on light or dark exposition. On the contrary, SOD activity in *Klmga2*Δ mutant cells (Figure 6A) resulted higher in light condition (10.9 ± 0.73 U mg^–1^ mg^–1^), while in dark condition it was very similar to wild type (6.4 ± 0.04 U mg^–1^). Differently, *Kl*Ctt1/*Kl*Cta1 activity (Figure 6B) in *Klmga2*Δ mutant cells was higher both in dark (12.0 ± 1.31 U mg^–1^) and light (13.8 ± 1.18 U mg^–1^) growth conditions compared to wild type (4.8 ± 1 and 4.7 ± 0.33 U mg^–1^ in dark and light, respectively).

We performed enzyme activities assays in the *Klmsn2*Δ strain too, since in *S. cerevisiae* Msn2 upregulates the cytosolic catalase gene *CTT1* (Hasan et al., 2002), which resulted strongly transcribed in *Klmga2*Δ mutant cells (Figure 5D). In the *Klmsn2*Δ strain, we found a significantly decrease of *Kl*Ctt1/*Kl*Cta1 activity (3.2 ± 0.38 U mg^–1^ and 3.5 ± 0.14 U mg^–1^ in light and dark condition, respectively; Figure 6B) in respect to wild type cells, suggesting that also in *K. lactis* Msn2 upregulates the activity of catalases. No significant changes of SOD activity compared to wild type were found in the *Klmsn2*Δ mutant.

### Effects of Light on Fad2 Desaturase Production

**FIGURE 7:**
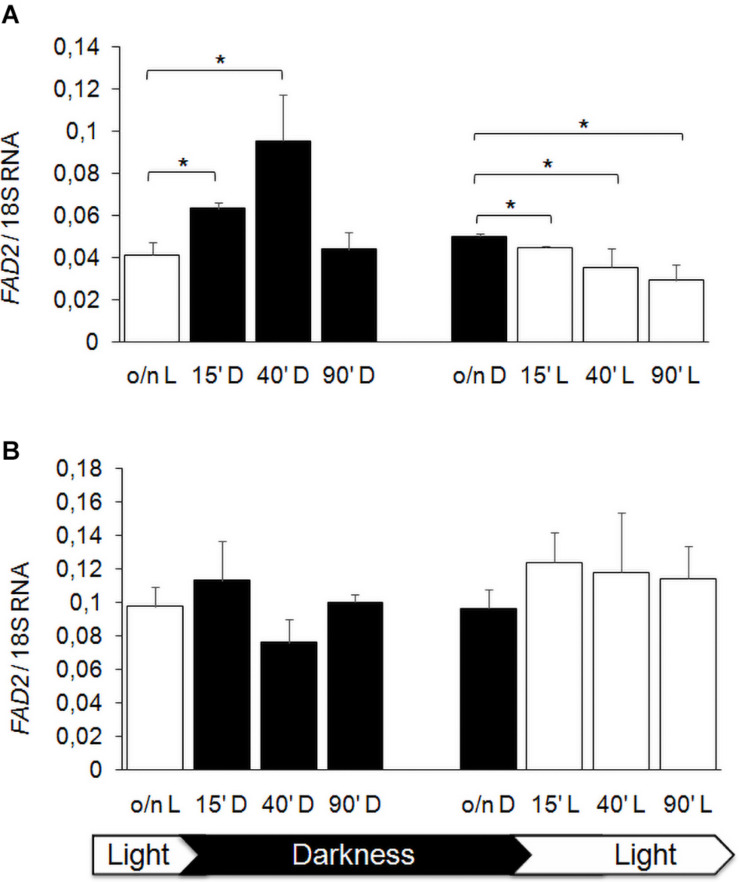
*FAD2* transcription after light and darkness shifts. Wild type **(A)** and *Klmga2*Δ **(B)** strain cells were grown overnight in light (o/n L) or darkness (o/n D) and then shifted to darkness (D) or light (L), respectively, for 15, 40, and 90 min before nucleic acid extraction and qRT-PCR. Values are averages of three independent determinations with SD. Transcription of ribosomal 18S gene was used as reference. * indicates *p* < 0.05.

Previous studies showed that FA composition of membranes is modulated by hypoxia in *S. cerevisiae* (Vasconcelles et al., 2001), by temperature and also by light exposure, especially in plant and bacterial cells (Kis et al., 1998; Hernández et al., 2011), as a stress adaptation response. We reported that *K. lactis* strain GDK/Klmga2Δ has an altered membrane composition and an inhibited hypoxic induction of the desaturase gene *FAD2*, which was the main FA desaturation target gene of *Kl*Mga2 (Ottaviano et al., 2015). We aimed studying a possible light regulation of desaturases expression in *K. lactis*, in particular, a *Kl*Mga2-dependent response to light. To this purpose, we first focused our study to the desaturase *FAD2* gene expression after light to dark and dark to light shifts. Results of qRT-PCR, reported in Figure 7, showed that the transcription levels of *FAD2* in wild type strain were influenced by light exposure. In detail, the shift from light to darkness caused a transient increase of transcription with a significant peak at 40 min from transition, while a progressive *FAD2* transcript reduction could be observed by shifting from darkness to light. Differently, the expression of *FAD2* in the *Klmga2*Δ mutant strain resulted substantially unchanged during light/dark shifts.

**FIGURE 8:**
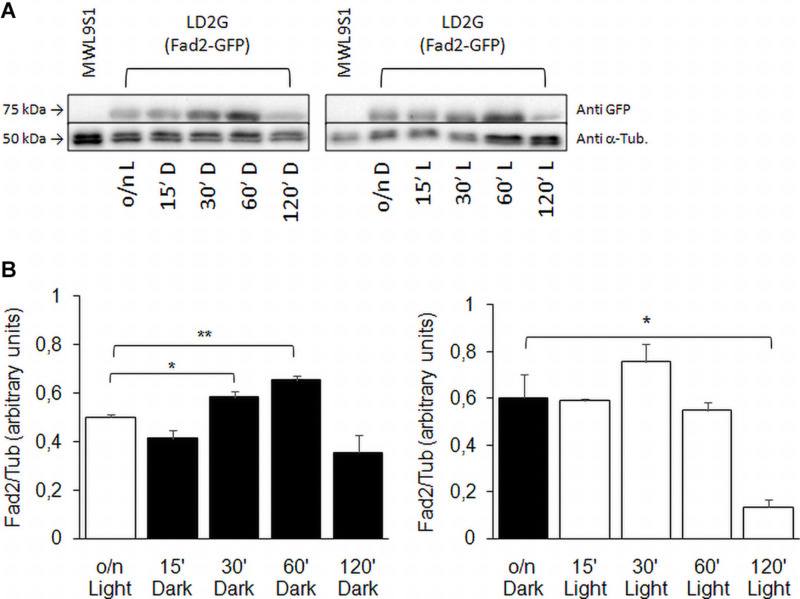
Fad2 enzyme production in wild type strain with *FAD2* fused to *EGFP*. **(A)** Shows Western blot against GFP-fused Fad2 protein, using strain LD2G (De Angelis et al., 2016). Cells were grown overnight in light or darkness and then shifted to darkness or light for 15, 30, 60, and 120 min. α-Tubulin detection was used as loading control. **(B)** Reports Fad2-GFP signal quantification with respect to α-Tubulin: values are averages of three independent determinations with SD. Significances: *p < 0.05; **p < 0.01.

The expression profile of *FAD2* in wild type strain was confirmed assaying Fad2 protein levels by Western blot (Figure 8). The shift from light to darkness caused a transient increase of Fad2 expression with a significant peak at 60 min from induction; from darkness to light, our results showed a Fad2 reduction after 120 min of light exposure.

### Effects of Light/Darkness Growth on FA Composition in Wild Type and *Klmga2*Δ Strains

**FIGURE 9:**
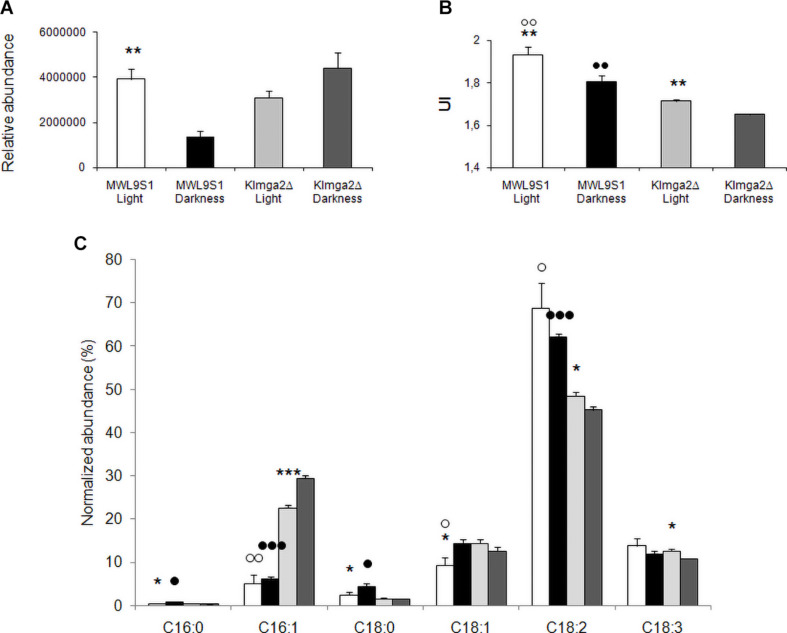
Fatty acid composition of the wild type MWL9S1 and *Klmga2*Δ mutant strain. Cells were grown in overnight light or darkness. **(A,B)** Report total fatty acid (FA) content (relative abundance with respect to 9-hydroxy octadecadienoic d_4_ acid, 9-HODE d_4_) and unsaturation indexes (UI) of the wild type and deleted mutant strain, respectively, with SD reported. **(C)** Reports amounts of palmitic (C16:0), palmitoleic (C16:1), stearic (C18:0), oleic (C18:1Δ9), linoleic (C18:2Δ9,Δ12), and linolenic (C18:3Δ9,Δ12,Δ15) acids (relative abundance with respect to HODE, normalized to the total amount of FAs). Results with SD are means of three independent determinations. Statistical relevance: stars = light vs dark, white dots = wild type vs mutant (light conditions), black dots = wild type vs mutant (dark conditions); one star or white/black dot = *p* < 0.05, two stars or white/black dots = *p* < 0.01 and three stars or white/black dots = *p* < 0.001.

Desaturases control the synthesis of unsaturated FAs and, consequently, composition of membranes. We performed FA analysis of wild type and *Klmga2*Δ mutant cells grown overnight in light or darkness (Figure 9). In the wild type strain, we observed a significant higher level of total FAs when cells were grown in light rather than in darkness, while in the mutant strain, FAs were high independently on light or darkness (values: Figure 9A). We calculated the Unsaturation Index (UI), a parameter of membrane fluidity: a low UI indicates a low fluidity of the membrane lipids due to a reduced percentage of unsaturated carbon–carbon bonds in FA molecules. Results in Figure 9B indicated a lower UI for the mutant strain with respect to the wild type, as also previously reported (Ottaviano et al., 2015), regardless on light (1.71 ± 0.005 vs. 1.93 ± 0.03) or dark (1.65 ± 0.004 vs. 1.81 ± 0.03) exposure. In addition, we have observed that UI was higher in both strains when cultivated in light condition as compared to darkness.

The detailed analysis of palmitic (C16:0), palmitoleic (C16:1), stearic (C18:0), oleic (C18:1), linoleic (C18:2), and linolenic (C18:3) acids is reported in Figure 9C. In the wild type strain, the growth in darkness resulted in an increase of saturated palmitic and stearic acids and of the mono-unsaturated (MUFA) oleic acid: the reduction of UI in darkness depended on the compensatory decrease of poly-unsaturated (PUFA) linoleic and linolenic acids. In the mutant strain we observed slightly different profile: the significant increase of palmitoleic acid was compensated by the decrease of C18 unsaturated oleic, linoleic and linolenic acids. The most relevant differences between the wild type and the mutant strains were significant increase of palmitoleic acid in the mutant strain, both in light and darkness growth (and oleic, but only in light), and the significant reduction of linoleic acid in the mutant strain, again in both light and darkness conditions.

**FIGURE 10:**
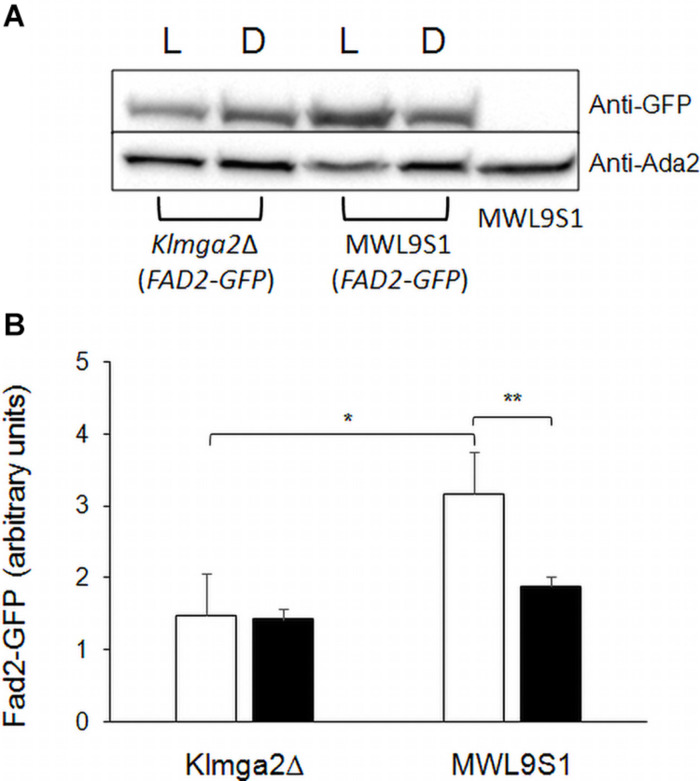
Fad2 production in *Klmga2*Δ mutant strain and wild type. **(A)** Shows a Western blot against Fad2-GFP protein, using extracts of strains LD2G and *Klmga2*Δ*/*Fad2-GFP grown overnight in light (L) and dark (D) conditions. Ada2 protein was used as loading control. **(B)** Reports a Fad2-GFP signal quantification of repeated Western blot experiments. White blocks represent Fad2-GFP in light and black blocks in darkness, respectively. SD are indicated. Statistical significance: *p < 0.05, **p < 0.01.

These changes might depend on different production of the desaturases Ole1 and Fad2 in the mutant strain. To verify this hypothesis, we determined the amount of Fad2 by Western blotting in the wild type and in the mutant strains grown in light and darkness. Results, reported in Figure 10, showed a significant reduction of Fad2 in the mutant strain with respect to the wild type, when grown under light stress, suggesting that, at least in light condition, the reduction of linoleic acid could be correlated to reduction of the corresponding biosynthetic enzyme.

## Discussion

Light is an ubiquitous and free source of energy. Light availability allowed the evolution of organisms endowed of biochemical systems able to capture and use this energy for biosynthesis. Also organisms lacking such molecules or proteins are subjected to light and are possibly set to respond to its exposure. Microorganisms like the unicellular yeasts *K. lactis* and *S. cerevisiae* do not have recognized light-responsive protein or photo-receptive molecules but are transparent to light and their metabolism and physiology might be influenced by light.

### Light Stress in *K. lactis*

Very few data, related to the light response in yeast, are available. In *S. cerevisiae*, the two regulatory factors Msn2 and Crz1 are environmental-stress regulators and a similar function has been recorded also for Msn2 in *K. lactis* (Barsoum et al., 2011). Msn2 and Crz1 are also light-dependent proteins in *S. cerevisiae*, translocated to nuclei under light exposition (Cai et al., 2008; Bodvard et al., 2011), suggesting a possible light-stress response dependent on the expression of specific target genes. Light signaling can be mediated in *S. cerevisiae* by the synthesis of hydrogen peroxide and the activity of PKA through the action of Pox1/Tsa1/Trx1 enzymes (Bodvard et al., 2017). All these elements are also present in *K. lactis* (*TPK1/2*: *KLLA0B07205g*, *TPK2*: *KLLA0D03190g*, *BCY1*: *KLLA0E04181g*, *POX1*: *KLLA0F09933g*, *TSA1*: *KLLA0B01628g*, *TRX1*: *KLLA0E16347g*), suggesting that a similar mechanism might be active also in this yeast.

In wild type *K. lactis* cells, light had no effects on growth rate. Differently, in the absence of *KlMGA2* gene, growth in darkness proceeded faster than in light, suggesting that that light could act as stressing element respect to growth and that *Kl*Mga2 could counteract light effect, protecting cells from adverse events. An altered membrane composition of the *Klmga2*Δ strain is a direct consequence of the reduced expression of *FAD2*, which is a main target of *Kl*Mga2 (Ottaviano et al., 2015). However, the deletion of *FAD2* gene did not affect light growth, suggesting that the differences in growth between light and dark conditions observed in the *Kl*mga2Δ strain should not be a direct consequence of the altered FAs membrane composition that characterizes the mutant strain.

### Mitochondrial Functions

We have previously reported that the deletion of *KlMGA2* causes altered mitochondrial morphology and respiration rate (Ottaviano et al., 2015). The recovery of normal mitochondrial morphology and the increase of respiration rate of the *Klmga2*Δ strain in the darkness confirm that *K. lactis* can sense light and that *Kl*Mga2 could play a role to mediate or attenuate phenotypes specifically correlated to respiration and/or mitochondrial functions in response to light. The deletion of *KlMSN2* did not caused sensitivity to the mitochondrial drug antimycin A (rag- phenotype), as in the case of *KlMGA2*. A slight sensitivity to the drug in darkness suggests an opposite regulatory effect of *Kl*Msn2, respect to *Kl*Mga2, in the mechanism of antimycin A resistance in light/dark conditions. The deletion of *KlCRZ1* did not cause evident phenotypes in the conditions explored in this work.

### Activation of *Kl*Mga2

Our results indicated that light or dark exposition did not induce the *Kl*Mga2 maturation process. However, the slight sensitivity to antimycin A, especially in darkness, of the constitutively active *mga2*ΔTM strain suggests a possible role of the full-sized membrane-bound *Kl*Mga2 form in contrasting drug activity. A detailed study of *Kl*Mga2 maturation and the physiological characterization of the strain harboring the *Kl*Mga2 truncated form, such as growth, oxygen consumption, and protein localization, will require further targeted investigations.

### Oxidative Stress

We have demonstrated in previous work (Santomartino et al., 2019a) that *Kl*Mga2 is involved in the response to oxidative stress and regulates chronological life span. It has been reported that light can induce ROS generation and oxidative stress in *S. cerevisiae* (Bodvard et al., 2017). In *K. lactis*, we found that the activity profiles of *Kl*Ctt1/*Kl*Cta1 and SOD enzymes and the transcriptional profiles of the corresponding genes were consistent with a general overactive ROS response in the absence of *KlMGA2* when the cells were exposed to light, especially as far as SOD expression was concerned. Our results also suggested a possible role of *Kl*Mga2 as downregulator of Msn2, because we observed high *Kl*Ctt1/*Kl*Cta1 activity in the absence of *Kl*MGA2, which was instead reduced in the absence of *KlMSN2*. In the latter case, the mechanism of action seemed to be independent on light/dark exposition.

### Lipids

FA composition of membranes determines their properties and functionality and depends on the expression FA biosynthetic enzymes, especially desaturases. The expression of yeast FAs desaturases is regulated by various environmental factors, including hypoxia, temperature, and the presence of specific chemicals. In addition, different yeasts are endowed with different desaturases (Santomartino et al., 2017). Another environmental factor that might influence membrane composition is light: it has been shown that the expression of the desaturase gene *FAD2* in plant depends on light exposure (Kargiotidou et al., 2008; Dar et al., 2017). Our study on the expression of *FAD2* desaturase gene in *K. lactis* indicated that *Kl*Mga2 mediates the transient light-dependent regulation of *FAD2* observed in wild type cells, suggesting a light-stress dependent modulation of membrane composition. Our results showed opposite light regulation of transcription of *FAD2* and *SOD1/2* or *CTT1* genes in the wild type and in the *Klmga2*Δ mutant, suggesting that light stress influenced differently ROS metabolism and PUFA biosynthesis in *K. lactis* and indicating the involvement of other specific elements in the *Kl*Mga2-dependent response mechanisms.

Major differences between *K. lactis* cells cultivated in light and in darkness were in the total amount of FAs and in the unsaturation index (UI). Light cultivation caused a consistent increase of cellular FAs and an increase of UI. Light also caused specific changes in the amount of individual FA species. As far as FA biosynthesis and accumulation were concerned, the absence of *Kl*Mga2 simulated a light stress-like status with abundant FAs content. On the other hand, light stress could be associated with increased membrane fluidity but this response was not dependent on *Kl*Mga2. It remains to be investigated the specific connection between light stress and high FAs content and high UI. For example, the high FAs content might derive from accumulation of triacylglycerol, due to metabolic imbalance, or increase of functional membranes, dependent on organelle proliferation. These hypotheses could be further investigated by assessing lipid droplets in light and dark conditions.

Interestingly, the amount of oxylipins (oxidized FA, Supplementary Figure 5) increased in dark conditions concomitantly to FA decrease in both strains. Especially 13-hydoxyoctadecenoic acid (13-HODE), an oxidation product of linoleic acid, increased in darkness (not shown). We can suggest that part of the loss of linoleic acid under dark conditions experiments by both strains could be partly due to the synthesis of oxylipins. This synthesis can be spontaneous, i.e., occurring from double bond oxidation by ROS, and enzymatically, i.e., catalyzed by lipoxygenases or dioxygenases.

## Conclusion

Results reported in this work indicate that the yeast *K. lactis* is responsive to light and that the regulatory factor *Kl*Mga2 has a role in this response. It has been shown that light induces oxidative stress in *S. cerevisiae* (Bodvard et al., 2017) and a similar activity could be envisaged also in *K. lactis* with the involvement of *Kl*Mga2, because of the effect of light on the detoxifying enzymes catalase and SOD. The deletion of *KlMGA2* gene has pleiotropic effects, suggesting that *Kl*Mga2, possibly because of its biochemical characteristics (Camponeschi et al., 2020), participates in various pathways: uncovering the detail of these pathways requires further investigation.

One of the most relevant pathway regulated by *Kl*Mga2 is the biosynthesis of FAs, and we showed here that also this pathway is influenced by light stress. The connection among light stress, membrane composition or functionality, and *Kl*Mga2 is highlighted by the fact that phenotypes of the *Klmga2*Δ strains, like growth rate, respiration rate, and mitochondrial morphology, are partially or completely suppressed either by darkness or by unsaturated FAs (Micolonghi et al., 2012; Ottaviano et al., 2015; Santomartino et al., 2019a). Finally, our findings open new perspectives on the role of light in the biology of organisms (apparently) deprived of light sensing proteins and on the role of lipids and membranes in the response to light stress.

## Data Availability Statement

The original contributions presented in the study are included in the article/ Supplementary Material, further inquiries can be directed to the corresponding author/s.

## Author Contributions

IC performed and conceived the experiments and wrote the manuscript. AM, MR, and CM performed the critical reading. MB performed the experiments and critical reading. MMB conceived the experiments, wrote the manuscript, and provided the funding. All authors contributed to the article and approved the submitted version.

## Conflict of Interest

The authors declare that the research was conducted in the absence of any commercial or financial relationships that could be construed as a potential conflict of interest.
