# Supplementary material for: Light-Stress Response Mediated by the Transcription Factor KlMga2 in the Yeast Kluyveromyces lactis
Source: Front Microbiol. 2021 Jul 14;12:705012. doi: 10.3389/fmicb.2021.705012 (PMC8317464; doi:10.3389/fmicb.2021.705012)
Supplement: Supplementary file 1 [file Data_Sheet_1.doc]

**Supplementary material**

**Table S1 -** List of oligonucleotides

| **N** | **Name** | **Sequence** | **Use** |
| --- | --- | --- | --- |
| 1 | CRZ1-DDK | ATGGATATTGACGATTATTTGAATGTCGATAGTCCCAGTGACATCGGTGCCAGCTGAAGCTTCGTACGCT | *Klcrz1::kanMX4* deletion cassette |
| 2 | CRZ1-UUK | GAATGGCATTTGTATTATTAGAGTAAGCGGACACTATTGGAATGTCATATGCATAGGCCACTAGTGGATC | *Klcrz1::kanMX4* deletion cassette |
| 3 | MSN2-DDK | ATGGCTCTGGGTCGGTATGAGTCTGGAAACCGAGGCTCTTATACTTCAGACAGCTGAAGCTTCGTACGCT | *Klmsn2::kanMX4* deletion cassette |
| 4 | MSN2-UUK | TGTATCTCATTTCCAATGTCACATCTGAAATATCGTAGATTATTTCAGTGGCATAGGCCACTAGTGGATC | *Klmsn2::kanMX4* deletion cassette |
| 5 | MGA2-URA3 fw | GGGCAATTACTATTAGATAGGAATAGTCGTGCTTTGCTGAAGTCAAGGGAGAGTGCACCATACCACAG | *Klmga2::URA3* deletion cassette |
| 6 | MGA2-URA3 rv | GATTCGACTATATACGGGCTTTGCACATAGATAAAAGGTCGGTTCAAGGAGTTTAGTATACATGCATTTAC | *Klmga2::URA3* deletion cassette |
| 7 | MGA2∆TM-fw | CTATCCAACTCTTAGTAGAATGTAAGGCCAACGTTACAGCAAGGGCAGACTCTAGATACCCATACGATGTTC | *Klmga2*∆TM-*3HA* fusion cassette |
| 8 | MGA2∆TM-rv | GATTCGACTATATACGGGCTTTGCACATAGATAAAAGGTCGGTTCAAGCGAATCGACAGCAGTATAGC | Klmga2∆TM-*3HA* fusion cassette |
| 9 | S3-fad2 | TTCAGAAACGTGAACAACGTTGGTGTCGGAACTGGTAAGAAGAAGAACCGTACGCTGCAGGTCGAC | *FAD2-GFP* fusion cassette |
| 10 | S2-fad2 | TGAATTGATGGAAACTAAAGAAACCAAGTTAATTGTTAGCTAGTTCAATCGATGAATTCGAGCTCG | *FAD2-GFP* fusion cassette |
| 11 | qCTA1-fw | GCTACGTGCAAGTGCATTTG | qRT-PCR |
| 12 | qCTA1-rv | GGTAATTACCCTTGGCGATG | qRT-PCR |
| 13 | qCTT1-fw | GCTATGTTCAGACGATGACC | qRT-PCR |
| 14 | qCTT1-rv | GAGAGAAAGCAAGTTGCTCG | qRT-PCR |
| 15 | qSOD1-fw | CCATGCATTCTCTGATGGAC | qRT-PCR |
| 16 | qSOD1-rv | CGAACGGTGTGGATATACTG | qRT-PCR |
| 17 | qSOD2-fw | GGGAAGCTTTCACCAAGATC | qRT-PCR |
| 18 | qSOD2-rv | GGGTATGAATCTCTCATCGG | qRT-PCR |
| 19 | qFAD2-fw | CTGACCAATGGTCCTTCGC | qRT-PCR |
| 20 | qFAD2-rv | TGAAGCAGGTCTAGCGTTGT | qRT-PCR |
| 21 | 18S-fw | CGGACTCCTTGATGATTCAT | qRT-PCR |
| 22 | 18S-rv | GATAGGGCAGAAATTTGAATG | qRT-PCR |

**FIGURE S1**

A


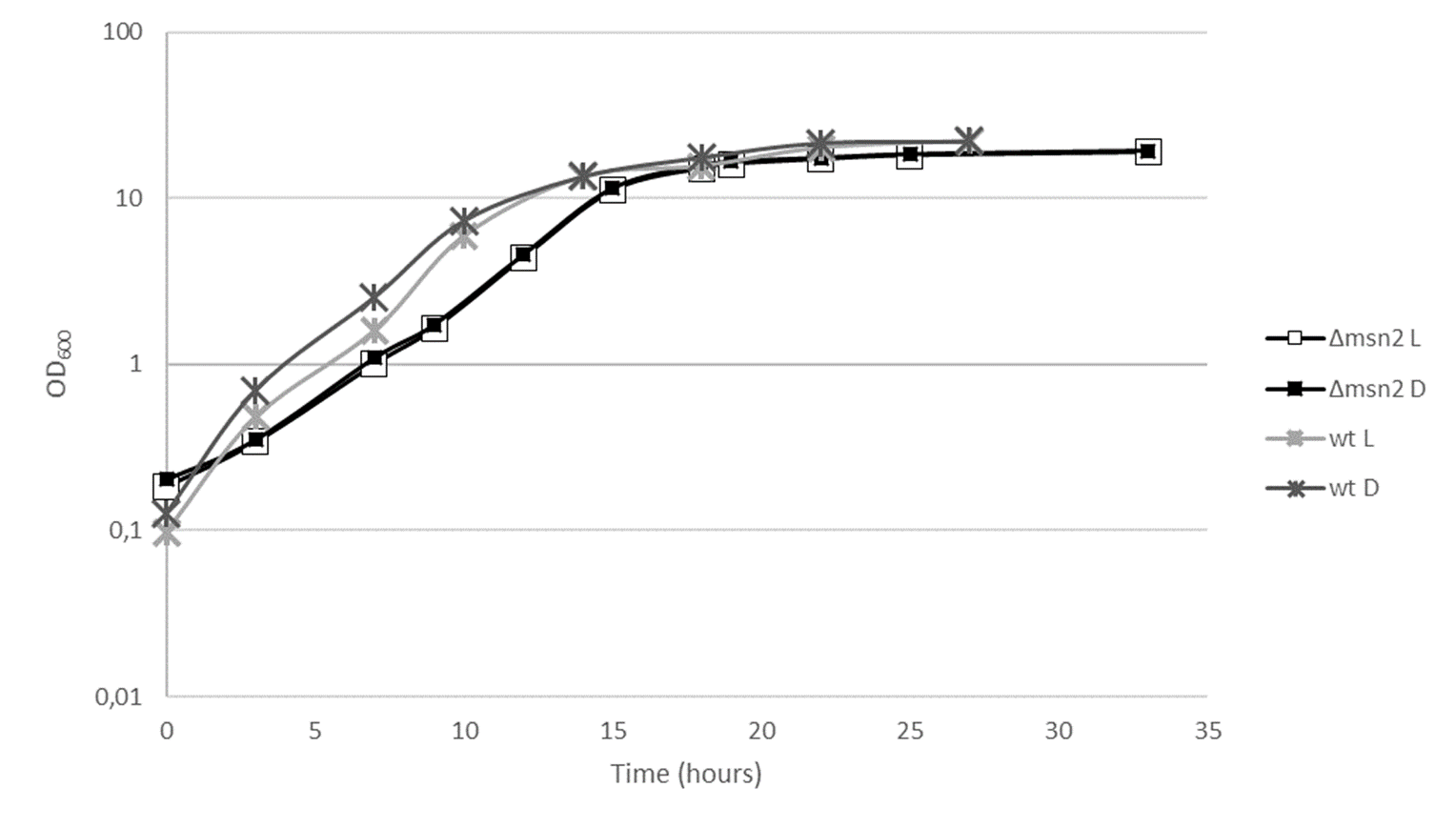


B


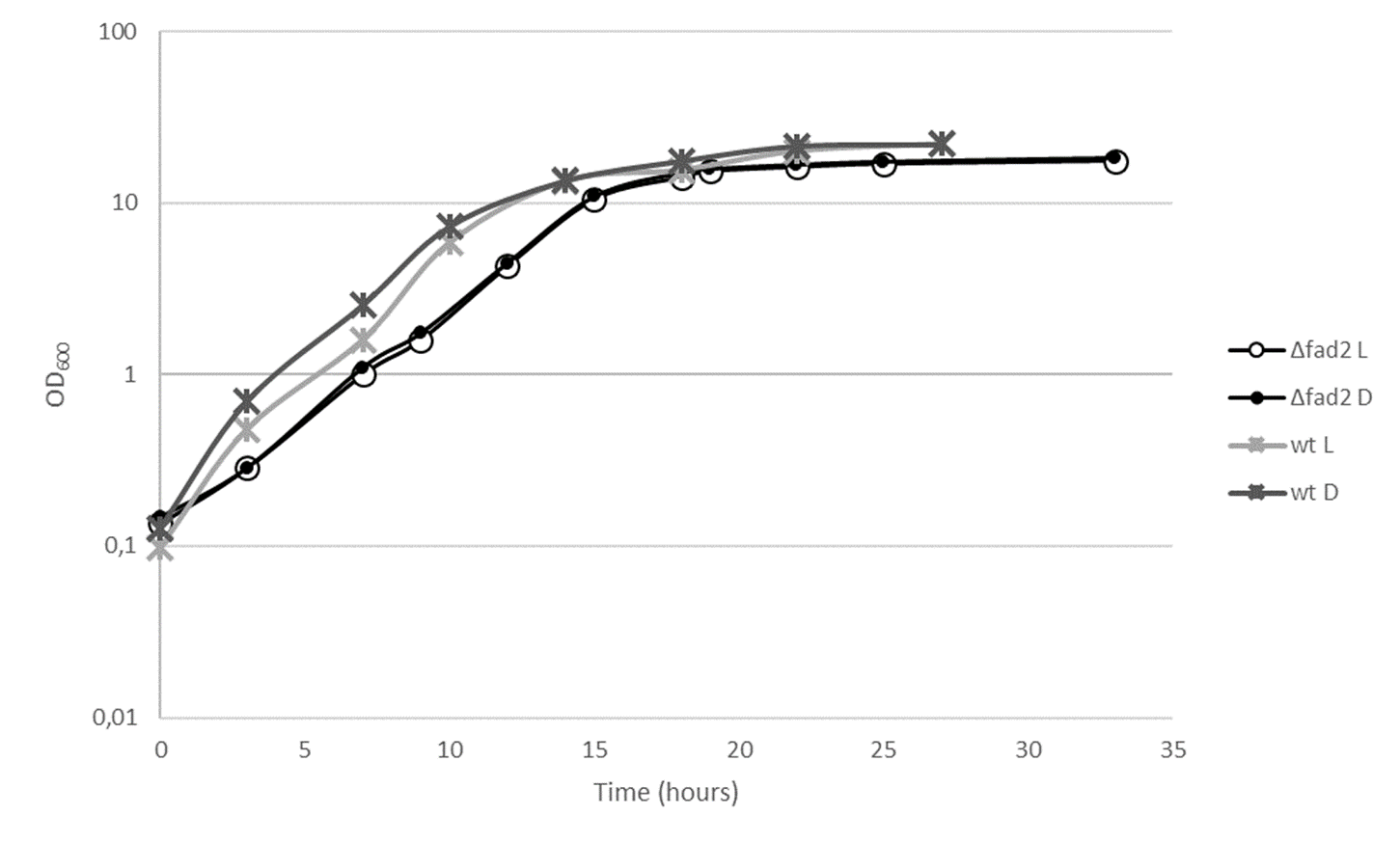


**Figure S1 – Growth curve of *Klmsn2*Δ and *fad2*Δ strains at light or dark exposure.** The growth in batch of the wild type (asterisk) and the mutant strains, *Klmsn2*Δ (squares, Fig. S1A) and *fad2*Δ (circles, Fig. S1B), is reported as optical density (OD_600_) over time (hours). Black/dark gray and white/light gray symbols refer to darkness and light cultivation, respectively. Growth medium was YPD.

**FIGURE S2**

A


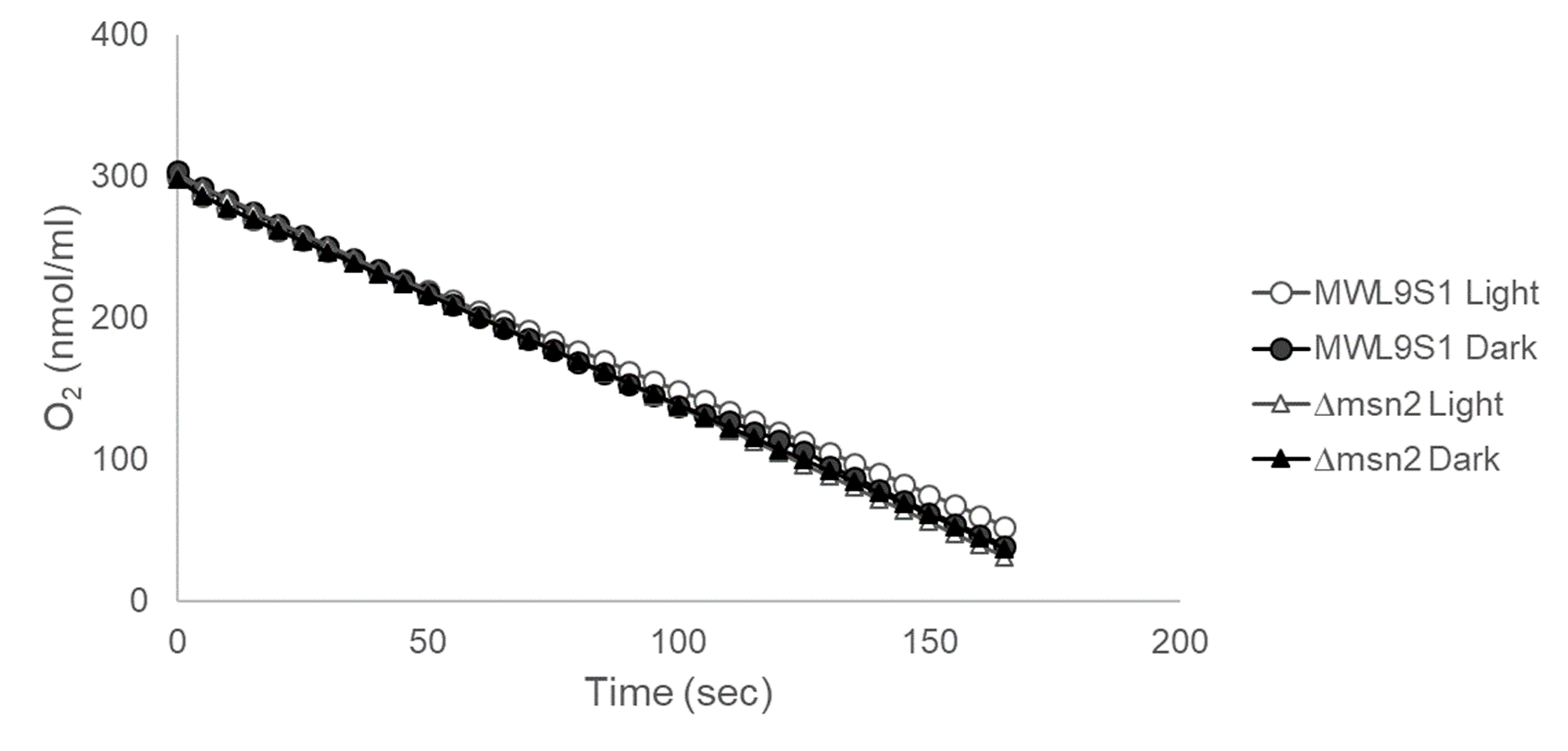


B


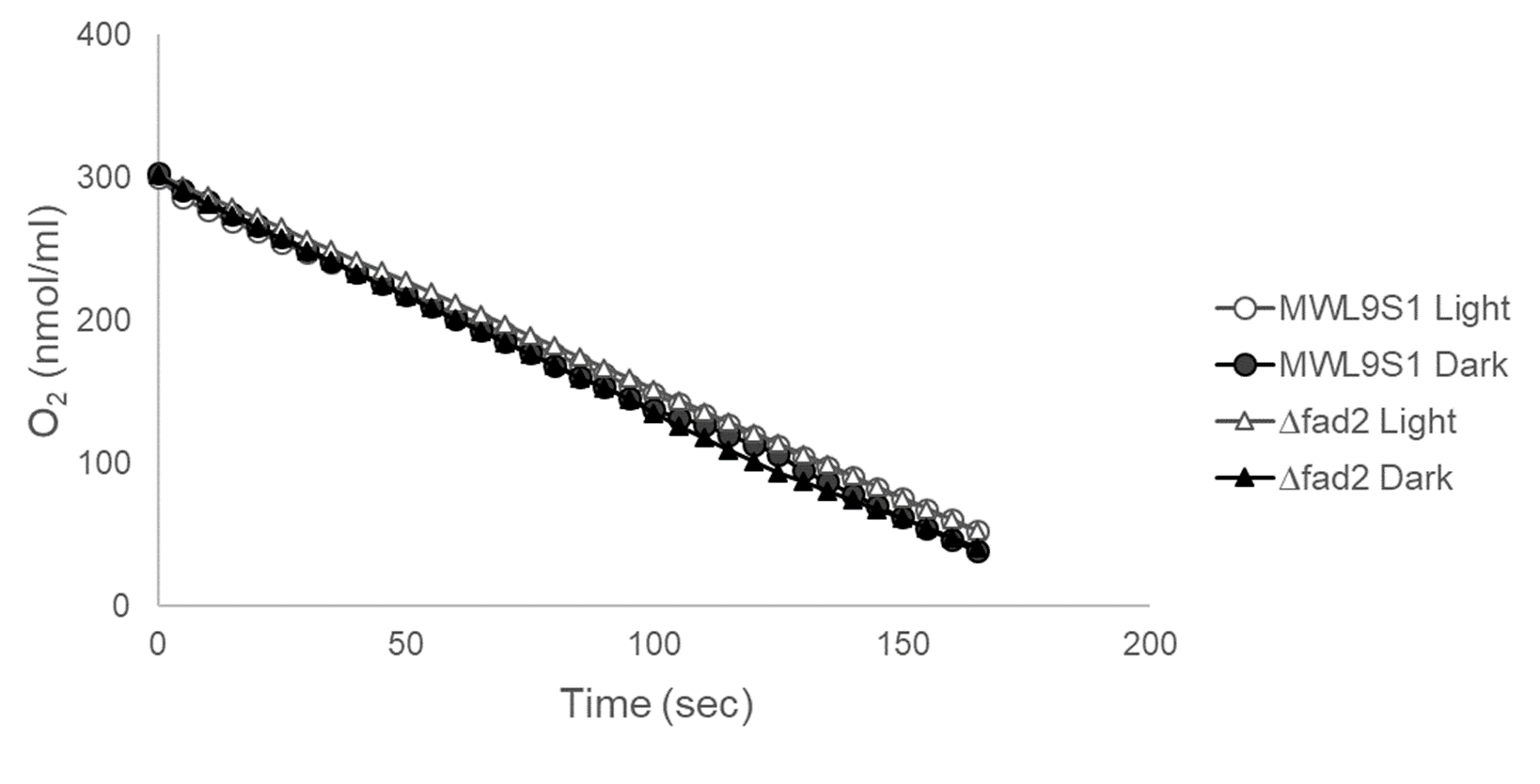


**Figure S2 – Oxygen consumption rates of wild type strain MWL9S1 and of mutant strains *Klmsn2Δ* and *fad2Δ*.** Respiration was determined as oxygen consumption rate (nmol/ml) by wild type (circles) and mutant strains (triangles; *Klmsn2*Δ, Fig. S2A and *fad2*Δ, Fig. S2B), grown in YPD. Black and white symbols refer to darkness and light cultivation, respectively.

**FIGURE S3**

**Figure S3 - Fluorescence microscopy analysis of *Klmga2*Δ strain.** Additional images of DASPMI staining of the mitochondrial network of the deleted strain grown in light condition.

**Figure S4**

**
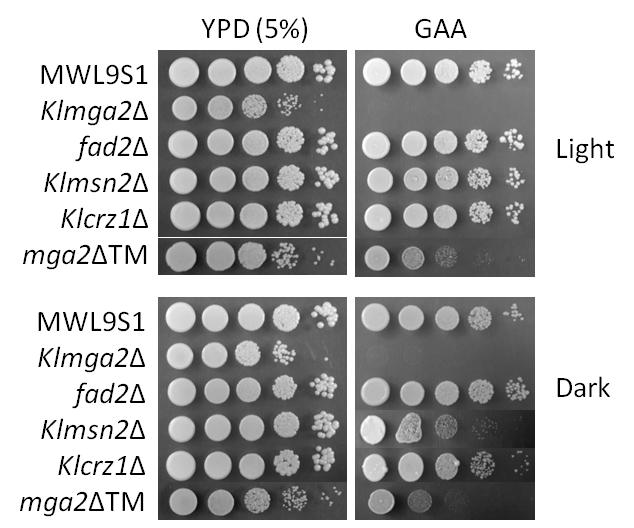
**

**Figure S4 - Growth on antimycin A medium of wild type and mutant strains.** Cultures grown in light and dark conditions in YPD medium were plated onto YPD plates (5% glucose) with or without antimycin A (GAA plates). Figure include data from the mutant strains *Klcrz1*Δ and *mga2*ΔTM.

**FIGURE S5**

**
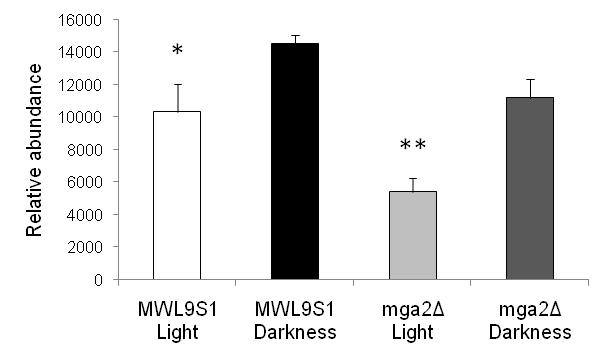
**

**Figure S5 - Oxidized fatty acids in the wild type strain MWL9S1 and in the mutant strain *Klmga2*Δ.** The total amount of hydroxylated fatty acids in cell grown in light or dark conditions is reported. FAs species determined were: 12,13-DiHOME, 13-HODE, 13-HOTrE, 13-HpODE, 9,10-DiHOME, 9-HODE, 9-HpODE and 9-oxoODE. Stars indicate significances of light vs darkness data: one symbol = p<0.05, two symbols = p<0.01.
